# Machine-based method for multiplex *in situ* molecular characterization of tissues by immunofluorescence detection

**DOI:** 10.1038/srep09534

**Published:** 2015-03-31

**Authors:** Dmitry Yarilin, Ke Xu, Mesruh Turkekul, Ning Fan, Yevgeniy Romin, Sho Fijisawa, Afsar Barlas, Katia Manova-Todorova

**Affiliations:** 1 Molecular Cytology Core Facility, Memorial Sloan Kettering Cancer Center, New York, NY 10065, USA

## Abstract

Immunofluorescent staining is an informative tool that is widely used in basic research. Automation of immunostaining improves reproducibility and quality of the results. Up to now, use of automation in immunofluorescent staining was mostly limited to one marker. Here we present tyramide signal amplification based method of multiple marker immunofluorescent detection, including detection of antibodies, raised in the same species, in tissue sections and cultured cells. This method can be beneficial for both basic and clinical research.

Immunohistochemistry (IHC) is one of the leading methods used to identify and co-localize antigens in cells and tissues, and has proven to be an effective and powerful diagnostic and research tool. While theoretically simple, the complex methods and protocols underlying IHC require validation and fine-tuning, detailed and complicated in design, to achieve accurate results. The complexity arises from the large diversity of cell-specific and tissue-specific molecular and macromolecular components and their modifications during sample fixation and processing^1^. Besides, variations due to “human factor” during manual staining may lead to unreliable results. Developments in automated staining technology have increased the feasibility of optimizing and standardizing protocols of immunostaining^2^,^3^. The use of commercial staining equipment greatly reduces variability of results, standardizes preparation steps and decreases workload in time-critical research and clinical settings. Most of the widely used machines allow automation of critical steps in staining: antigen retrieval, blocking, incubation, and detection. Thus, automation increases accuracy and minimizes the risk of human error. In addition, the reagents specifically optimized for these machines eliminate uncertainty in reagent choice, improve reproducibility outcomes and allow detection not only of proteins but also nucleic acids^4^.

Despite the advancements, most of the applications in automated IHC staining are limited to one or two markers for the same sections and are solely chromogen based. IHC staining using more than one primary antibodies is hampered by the restricted availability of compatible chromogenes and their stability. One should be extremely cautious to choose chromogenes that can be spectrally separated, when colors overlap due to close proximity of target molecules. Immunofluorescent (IF) staining overcomes this limitation by the availability of different wavelength fluorophores as detectors. In this case, staining is limited by antibody compatibility and by the ability of the microscope to accurately detect the specific fluorophores. Our laboratory has developed protocols that utilize the consistency of automated machine-based staining to perform reliable and highly reproducible single, double, triple and quadruple immunofluorescent (IF) staining of sections of both frozen and paraffin embedded fixed tissues. We achieved this by applying specific blocking and saturation steps (see Methods) and incorporating tyramide signal amplification within the automated staining procedure.

Tyramide Signal Amplification (TSA) is based on the ability of horseradish-peroxidase (HRP) to catalyze the deposition of large amounts of tyramide around the antigen-antibody complex. This phenomenon was first observed in the late 1950s^5^, but only decades later was applied for amplification of signal in immunoassays (ELISA and Western blot)^6^. The principle of reaction was then adapted to immunohistology^7^ and *in situ* hybridization^8^,^9^ to increase the sensitivity of detection system. Without such amplification, limited presence of the target molecule often renders the signal undetectable. While the TSA procedure is used in IHC, it is especially pertinent in IF staining. Since the amplification does not alter the relative variation in expression levels, the fluorescence level corresponds to the relative target antigen level. In other words, TSA amplification in IHC staining brings signal to detectable levels, while TSA amplification in IF staining not only boosts the signal, but reflects relative levels of target expression in the tissue. By combining this characteristic of the TSA protocol with the consistency of automated staining results, we are able to reproducibly perform successful IF experiments.

Characterizing co-expression and co-localization of multiple antigens is an important and often-used methodology in research as well as in clinical settings. However, reliable multiple-marker IF staining can be difficult to achieve. The specificity of each antibody must be validated in single staining using proper controls and must be retained when multiple antibodies are applied. Generally, antibodies raised in different species are used to prevent cross-reactivity. However, it is not always possible to find optimal antibodies of interest made in different species. Even if such antibodies are identified, achieving successful detections is not guaranteed.

Methods for double staining with antibodies derived from the same species have been published: 1) Adjacent thin sections are stained separately and images are superimposed^10^,^11^. 2) Primary antibodies of a particular isotype are detected with secondary antibodies, specific only for that specific isotype^12^. 3) Primary antibodies are directly conjugated to fluorophores, enzymes or haptens^13^,^14^,^15^. 4) Saturation of epitopes in double IF or IHC to prevent non-specific binding of antibodies of the same species was done with fluor- or respectively HRP- or Alkaline phosphatase-conjugated IgG (Fab) used as secondary antibodies^16^,^17^,^18^. 5) Tyramide amplification combined with conventional detection was used in double IF with frozen skin sections^19^. Unfortunately, all of these methods, except TSA-based, either have limitations or simply do not work under automated staining conditions.

In our laboratory we discovered that combining consecutive staining method with TSA-based detection allows use of multiple antibodies, including antibodies raised in the same species, in automated staining. Here, we present examples from a small subset of multi-channel and same-species fluorescent stainings of paraformaldehyde and formalin fixed paraffin-embedded sections of human and mouse tissues. To our knowledge, this is the first published demonstration of successful and reliable machine-based automated IF detection of multiple antigens.

## Methods

Adult mouse tissues (liver, testis, ovary and spleen) and E13.5 mouse embryos were fixed with freshly made 4% paraformaldehyde (PFA) in PBS (Sigma Aldrich) overnight at 4°C, processed with Leica ASP6025 tissue processor (Leica Microsystems), embedded in paraffin and sectioned at 5 μm. Human tumors were donated by the Pathology Department without any unique patient identifiers except diagnosis. Human tissues were fixed in 10% Neutral Buffered Formalin (NBF), unless otherwise indicated, processed with Leica ASP6025 tissue processor (Leica Microsystems), embedded in paraffin and sectioned at 5 μm.

All experiments were performed in accordance with approved guidelines established by MSKCC IACUC and IRB. The protocol #01-11-026 for mouse experiments was approved by MSKCC IACUC. Human tissues were used according to the research exempt from IRB/PB review SOP# FO-303 (Part 3.4).

The IF staining was performed using Discovery XT processors (Ventana Medical Systems). Sections were deparaffinized, conditioned and the antigens were retrieved with proprietary buffers, EZPrep and CC1 (Ventana Medical Systems). Slides were blocked for 30 minutes with peptide-based blocking reagent Background Buster (Innovex). Primary antibodies were applied at optimized concentrations previously determined on control tissues (see Table 1). Sections were incubated with primary antibody, followed by 60 minutes incubation with biotinylated secondary antibodies (Vector Laboratories) against immunoglobulins of the primary antibody source species at 7.5 μg/ml in PBS with 2% BSA and 1.5% normal serum of secondary antibody source species. The detection was performed with Streptavidin-HRP D (DABMap kit, Ventana Medical Systems), followed by incubation with one of the following Tyramide Alexa Fluors (Invitrogen): 488 (cat# T20922), 546 (cat# T20933), 568 (cat# T20914), 594 (cat# T20935) or 647 (cat# T20936) prepared according to manufacturer instruction with predetermined dilutions. Slides were counterstained with DAPI (Sigma Aldrich, cat# D9542) at 5 μg/ml in PBS (Sigma Aldrich) for 10 minutes, mounted with Mowiol (Calbiochem) and kept at −20°C.

The stainings were performed consecutively. The detection for each marker was completed before application of next antibody. The best sequence of antibodies for multiple staining was determined for each combination. When multiple staining was done with primary antibodies raised in the same species, tissue sections were “saturated” by re-incubation with the primary antibody they were just stained with, for 1 hour at room temperature in the dark, followed by washing in reaction buffer (Ventana Medical Systems). The experiment continued with the next primary antibody. In some cases slides were fixed in 4% PFA in PBS for 10 minutes between the stainings to stabilize the IgG complex of the first staining and prevent non-specific binding.

The slides were imaged at our Facility using Axioplan2 (Zeiss) with Axiocam, Leica SP5 confocal system (Leica MicroImaging), or Perkin Elmer/3DHISTECH Pannoramic Digital Slide Scanners. Z-stacks obtained from confocal imaging were visualized using Imaris (Bitplane).

## Results

**Figure 1:**
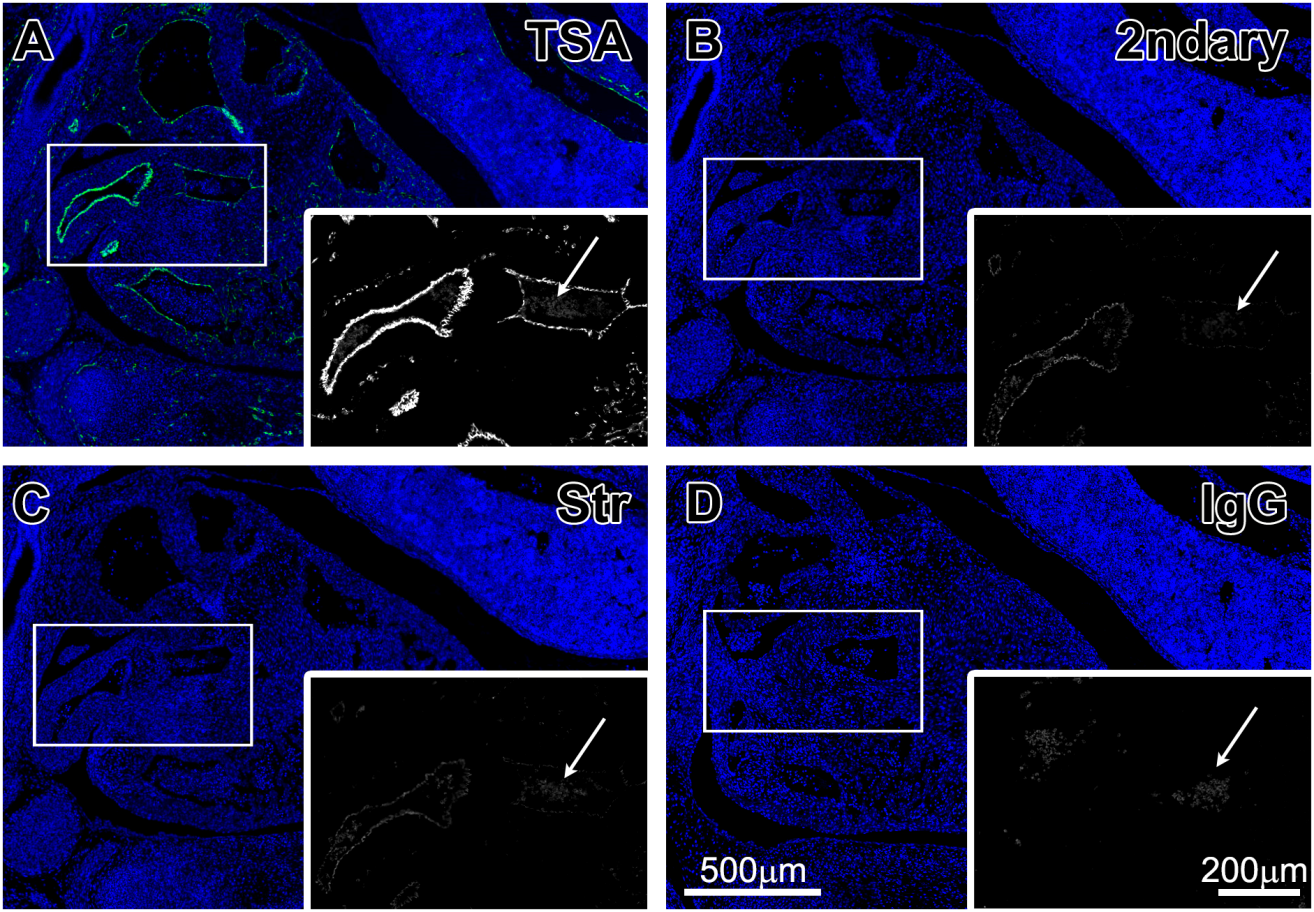
CD31 expression detected with different detection systems. Paraformaldehyde fixed, paraffin embedded 5 μm sections of E13.5 mouse embryo were stained with CD31 primary antibody (see Methods for details) and nuclei were stained with DAPI. Panel A shows detection with biotinylated goat anti-rat secondary antibodies followed by Streptavidin-HRP and TSA- Alexa488 amplification. Panel B shows detection with goat anti-rat secondary antibodies directly conjugated with Alexa488. Panel C shows detection with biotinylated goat anti-rat antibodies followed by Streptavidin-Alexa488. Panel D shows staining with isotype control IgG detected with biotinylated goat anti-rat antibodies, followed by Streptavidin-HRP and TSA-Alexa488. The inset in each panel represents a closer view of the indicated region, with CD31 signal showing in grayscale. White arrows point to autofluorescence emitted by the eurethrocytes, which persists in all staining methods.

We established a reliable and reproducible method of automated multiple marker IF detection on the Ventana Discovery XT instrument. The method employs the use of biotinylated secondary antibodies, Streptavidin-HRP conjugate and tyramide-fluorophore conjugate to detect and amplify the signal. Introduction of this method allows the sequential detection of multiple markers even when primary antibodies are raised in the same species. Earlier experiments, with other standard methods of fluorescent detection, produced suboptimal or even negative results. Only TSA-based system provided reliable and reproducible results. In Figure 1, we present the comparison of three detection methods: biotinylated secondary antibodies plus Streptavidin-HRP followed by TSA-Alexa 488 (Panel A); secondary antibodies conjugated with Alexa 488 (Panel B); and biotinylated secondary antibodies plus Streptavidin-Alexa 488 (Panel C), used to stain CD31 in mouse 13.5 embryo. One can appreciate that in cases of strong CD31 staining, detectable signal is present in all detection methods (although at very different levels), whereas in smaller blood vessels with lower expression levels of CD31, the signal is only detected with TSA-based method. We have numerous similar results for manual and automated stainings of both frozen and paraffin embedded tissues.

We applied this method to a wide range of tissues from different species (mouse, human, rat, rabbit, fish, Drosophila). In this article we present only a few examples of successful multiple stainings. Spleen is one of the tissues that is rather complicated for staining, as it is part of the immune system and contains many types of cells involved in the immune response. We used several combinations of markers to characterize the immune response in spleen.

**Figure 2:**
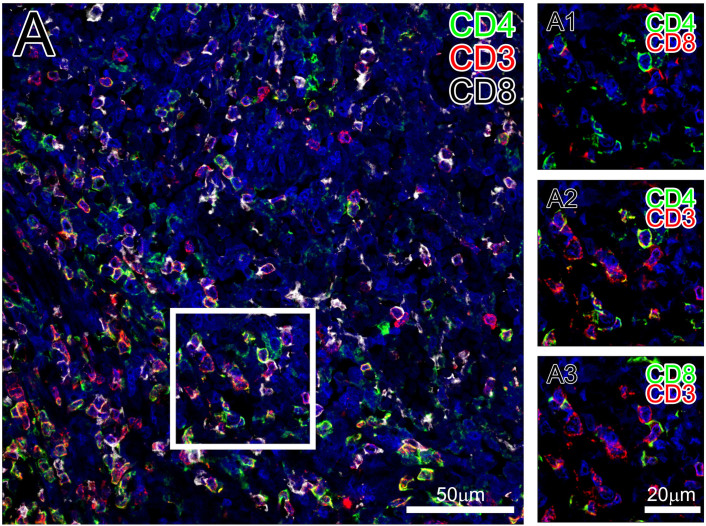
Immunofluorescent detection of T cell subpopulations in human spleen sections. Formalin fixed, paraffin embedded human spleen sections were stained with CD4, CD8 and CD3 antibodies (see Methods for details) and nuclei were stained with DAPI. Panel A shows an overview of the tissue area. Detailed views of the indicated subregion are in Panels A1-3: A1 reveals mutually exclusive CD4 (green) and CD8 (red) positive T cell subpopulations, A2 represents CD4 (green)/CD3 (red) double positive subpopulation and A3 depicts CD8 (green)/CD3 (red) double positive T cells.

**Figure 3:**
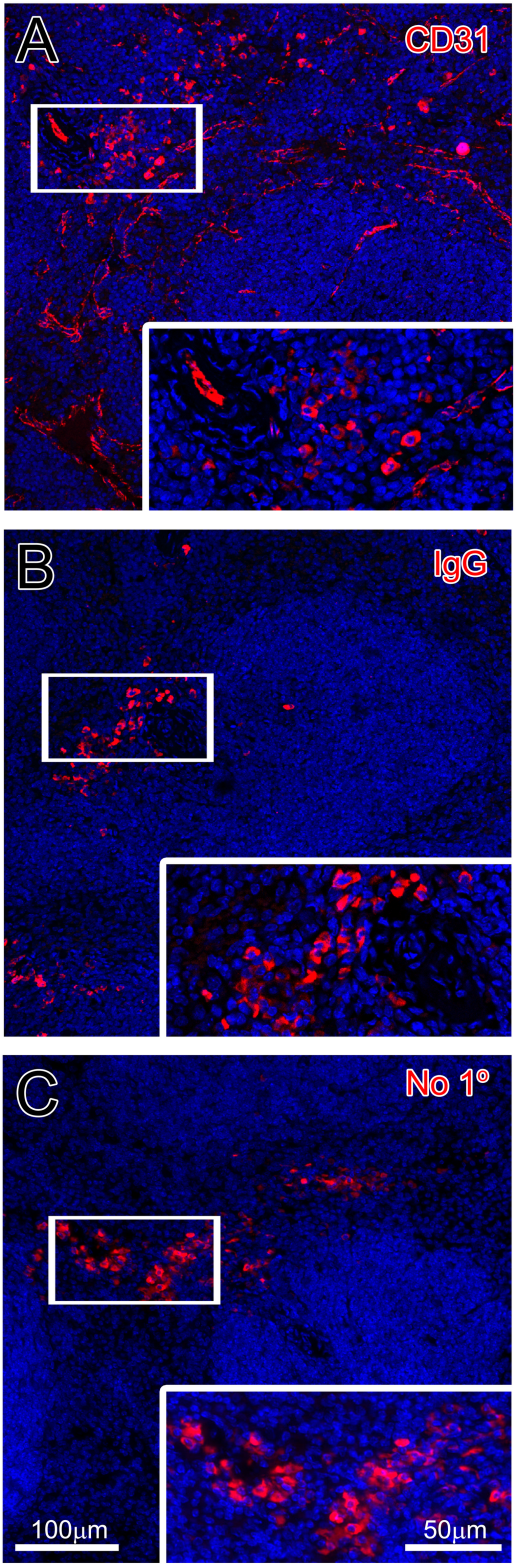
Example of presence of “plasmacytes” in mouse spleen staining with CD31, isotype control and no primary antibody control. Panel A shows the staining of blood vessels with CD31 along with non-specifically stained cells (red), Panel B represents just the non-specific staining (red) with isotype control (rat IgG) and in Panel C same “plasmacytes” are observed in staining that skips primary antibodies or isotype control.

In Figure 2, we present the staining of human spleen sections with CD4 antibody (mouse) in green, CD3 antibody (rabbit) in red, CD8 antibody (mouse) in white and DAPI in blue. CD4 and CD8 antibodies were used to distinguish the two major subpopulations of T cells that are CD3-positive. DAPI was used to stain cell nuclei. One can appreciate that the markers for two subpopulations – CD4 and CD8 – are distinct and appear to be expressed on different cells (Panel A1), whereas each subpopulation-specific marker is coexpressed with CD3 (Panel A2 for CD4/CD3 and A3 for CD8/CD3). Sequential IF staining with immune markers were also successfully performed using mouse spleen sections. However, when mouse/rat antibodies were used to stain mouse spleen, in addition to the specific signals, non-specifically labeled cells were frequently present (Figure 3). We consider those cells plasmacytes, rich with immunoglobulins, thus always stained with the secondary anti-mouse/rat antibodies. The same cells were also detected when isotype control IgGs were used instead of primary antibodies or in case when no primary antibodies were used (Figure 3).

**Figure 4:**
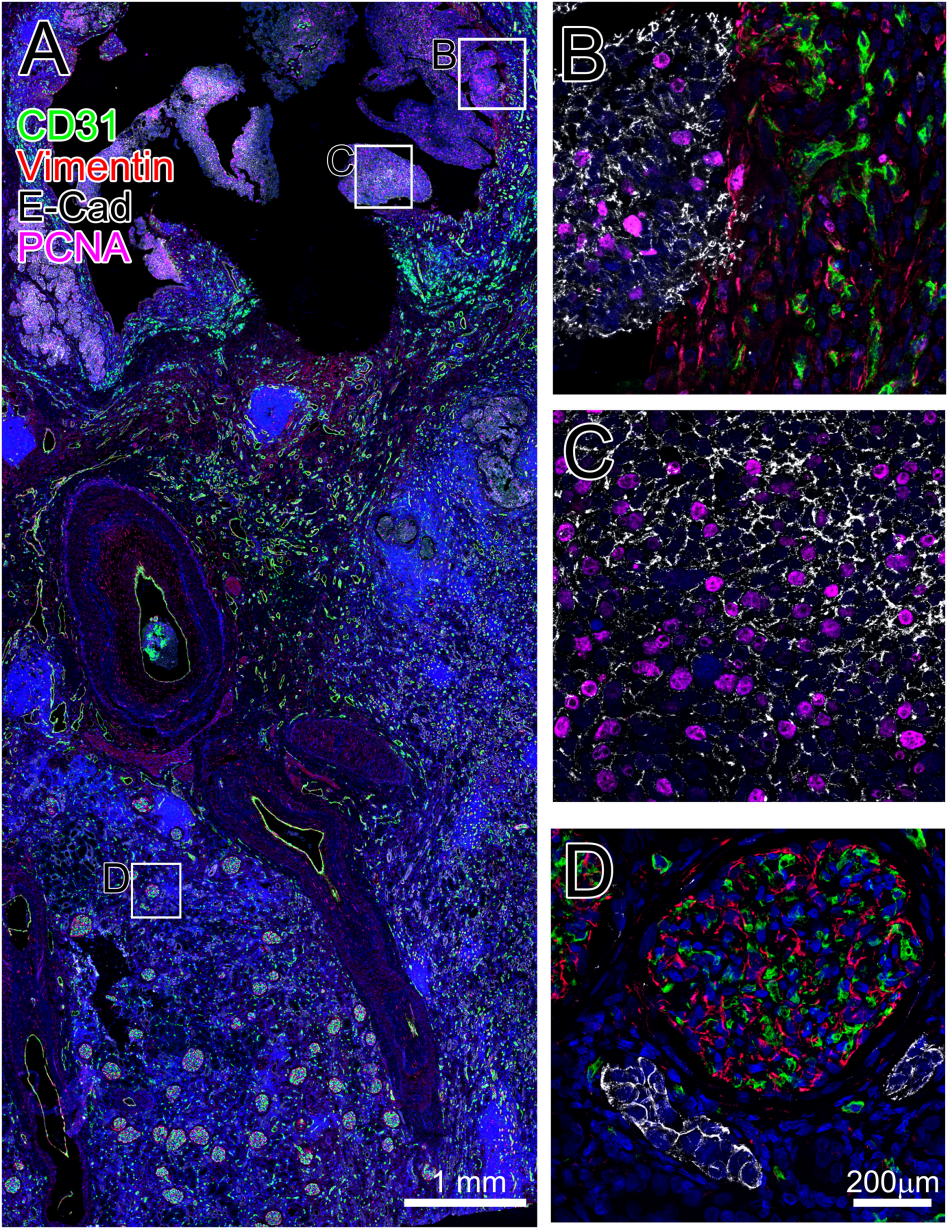
Quadruple staining of human kidney with tumor. Formalin fixed, paraffin embedded human kidney tumor sections were stained with CD31, Vimentin, E-Cadherin and PCNA antibodies (see Methods for details) and nuclei were stained with DAPI. Panel A shows a large area of the section that includes both “healthy” tissue and tumor areas. Panels B–D are magnified views of regions marked in panel A to emphasize patterns of staining for CD31 (green), Vimentin (red), E-Cadherin (white), PCNA (magenta) and DAPI (blue).

In another example, we present data from a successfully performed quadruple immuno-staining using the TSA method, shown in Figure 4. Human kidney tumor is stained for CD31 (mouse antibody) in green, Vimentin (guinea pig antibody) in red, E-Cadherin (mouse antibody) in white, and PCNA (mouse antibody) in magenta plus DAPI in blue. A representative fragment of the tissue section shows that none of these markers are co-localized and the signals are strong. Panels B, C and D reveal staining details of several regions of the tissue in higher magnification. As in any sequential antibody staining protocol, the sequence of antibodies used can greatly affect the quality of specific staining and levels of background. Importantly, the appropriate sequence could be specific for any combination of antibodies and must be determined by trial and error.

**Figure 5:**
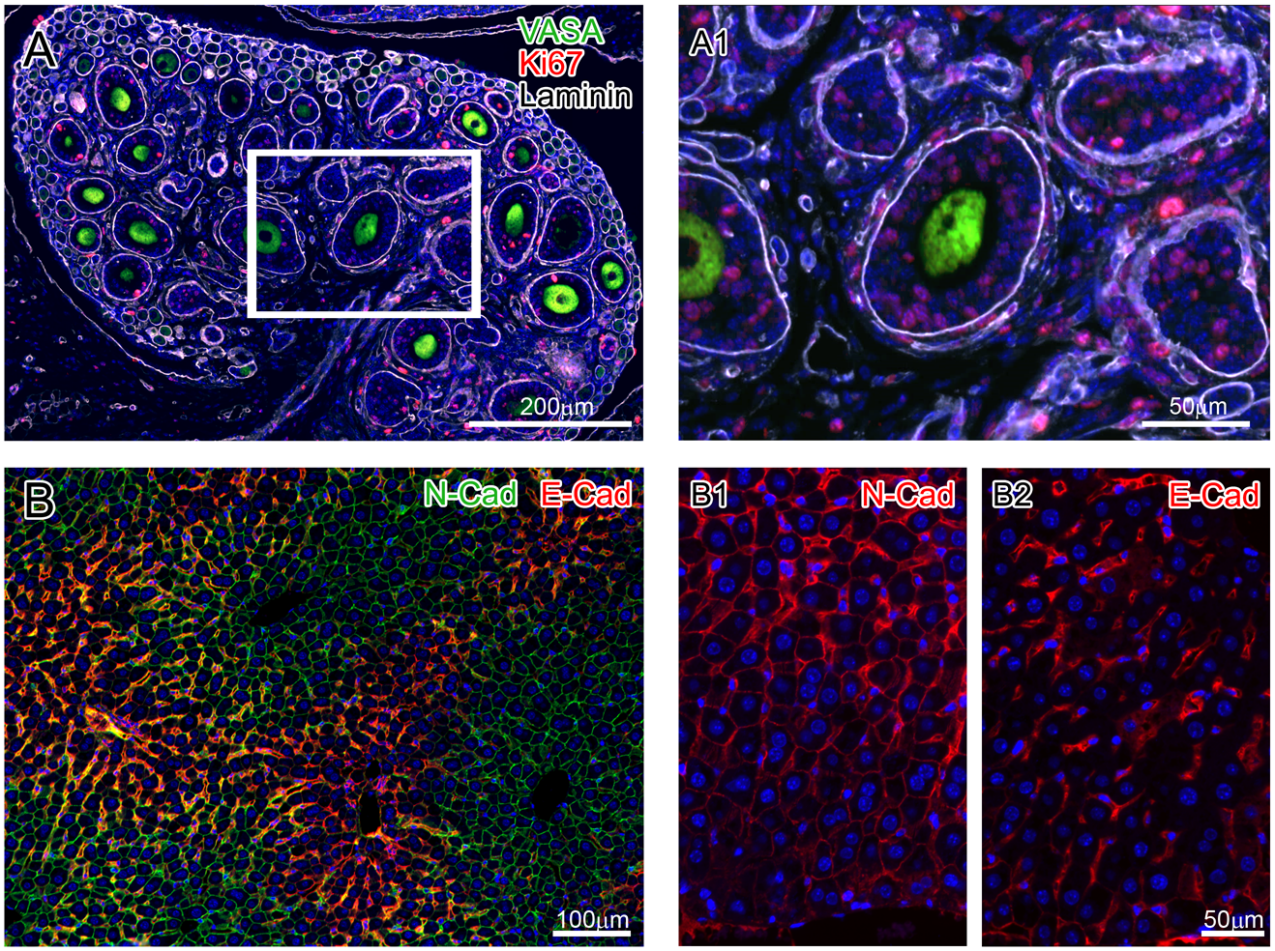
Examples of staining with several antibodies raised in the same species. Panel A shows a section of juvenile mouse ovary, stained with VASA (green), Ki67 (red) and Laminin (white) antibodies (see Methods for details), all of them raised in rabbit. Panel A1 is used for closer view of the staining pattern. Panel B depicts a section of adult mouse liver stained with two antibodies raised in mouse, N-Cadherin (green) and E-Cadherin (red). Panels B1 and B2 show single staining patterns for N-Cadherin and E-Cadherin respectively, both performed with red fluorophore, to emphasize that there is no cross- talk and the pattern of staining is well preserved in the double IF, independently of two different fluorophores used.

We have many examples of successful staining of different tissues combining antibodies from the same or different species and stained in different sequences (Supplemental Table 1). Figure 5 includes examples of multiple staining of mouse tissues with the antibodies derived from the same species. Panel A shows mouse ovary stained with three rabbit antibodies –VASA, Ki67 and Laminin. Panel B shows mouse liver stained with two mouse antibodies –E-Cadherin and N-Cadherin. Each of the combinations was tested several times and each staining included single marker controls as well as non-immune isotype controls. In addition, each marker was previously tested and validated by immunohistochemical staining with DAB detection.

**Figure 6:**
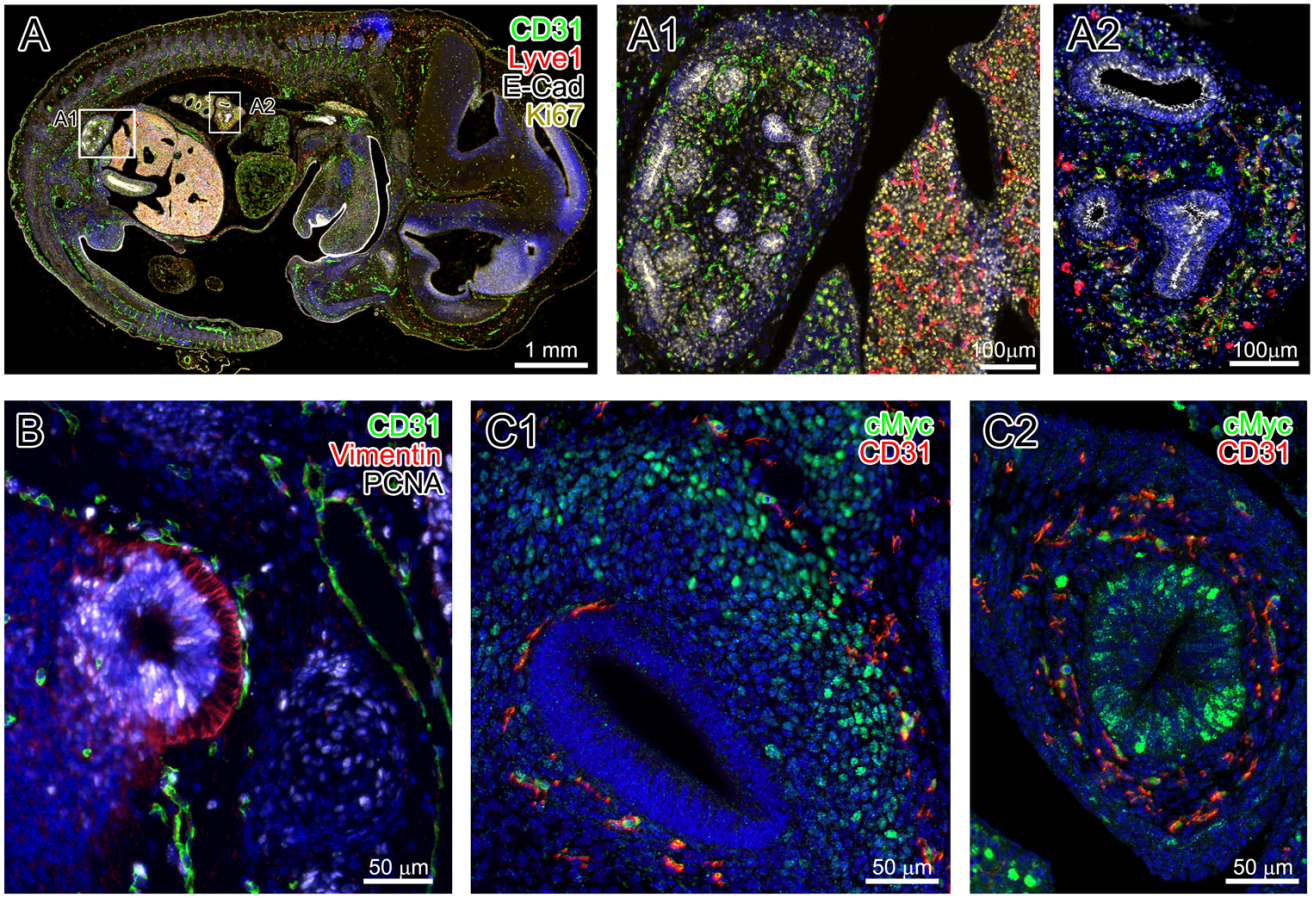
Examples of multiple staining in E13.5 mouse embryo. Panel A shows the mouse embryo stained for CD31 (green), Lyve1 (red), E-Cadherin (white) and Ki67 (yellow). Panels A1-2 are close up views of kidney and liver (A1) and lung (A2). Panel B includes olfactory lobe with CD31 (green), Vimentin (red) and PCNA (white) antibodies. Panel C1- a section through the cochlear and C2, the midgut, staining for c-Myc (green) and CD31 (red).

Human tissues presumably have higher rate of specificity, as the antibodies against human antigens are more vigorously tested and more readily available. However, more frequently, we stain embryonic and adult mouse tissues that pose greater risk of non-specific binding of antibodies. Figure 6 shows examples of successful multi-colored stainings of mouse E13.5 embryo. Panel A represents an overview of a paraffin section through a E13.5 embryo, as well as detailed views of staining with four markers –CD31 (rat antibody), a blood endothelial cell marker in green; Lyve 1 (goat antibody), for lymphatic and specialized blood endothelia, in red; -E-Cadherin (mouse antibody), epithelial cell marker, in white; and Ki67 (rabbit antibody), a marker for proliferation, in yellow and DAPI in blue. Yet again, one can see different structures outlined by the corresponding markers and the dividing cells (Ki67) in relation to those structures. Panel B shows an example of triple marker staining –CD31 (rat antibody) in green; Vimentin (guinea pig antibody), for intermediate filaments in mesenchymal cells, in red; and PCNA (mouse antibody) for proliferating cells in white, in mouse embryo. Panel C shows an example of double staining of embryo section with anti-c-Myc (mouse antibody) to assess differential anabolic activity, in green; and CD31 (rat antibody) in red.

In sum, our data show how powerful automated immunofluorescence detections can be and how much information it can bring to both clinical and basic research.

## Discussion

In this report, for the first time we present the method of automated IF staining with multiple markers in the same tissue sections. Furthermore, our protocol allows researchers to use antibodies raised in the same species. To prevent non-specific binding of the second primary antibodies to unbound epitopes of secondary antibodies, we perform “saturation” with primary antibodies, used in the first staining (normal IgGs can also be used). We utilize the TSA detection system that involves biotinylated secondary antibodies, subsequently labeled with Streptavidin-HRP and amplified with tyramide-fluorophore conjugates. When IHC staining is performed manually, this method is the best for detection of low-expression targets^19^,^20^,^21^,^22^. For an automated immunofluorescence protocol, TSA is the only method that produces reliable results (see Figure 1). In our hands, all other methods have resulted in either low signal, or no staining at all (see Figure 1). It is essential to note that the concentrations and incubation times of primary antibodies used for the IF stainings are the same as we use for chromogen-based IHC stainings. It is also important, that TSA-based method of IF detection, as opposed to TSA amplification in IHC detection, allows relative quantification of the signal levels.

Although in our laboratory we use Ventana Discovery machines, this approach could be adopted for any open automated platform that can utilize HRP-based detection. In a testing of a Leica Bond RX machine, we were able to perform IF staining of paraffin sections using open protocols and the same reagents we used for staining on the Ventana machine.

While one may take advantage of the reproducibility and convenience of automated staining, obstacles that befall any immuno-staining – manual or automated – are still present. For example, some antibodies are simply incompatible with one another: certain combinations of antibodies will unavoidably give non-specific staining or cross-reactivity. It is possible that the antibodies recognize similar fragments of antigens, or some tissue components are “sticky” and attract antibodies non-specifically. Automation and signal amplification with tyramide does not alleviate all problems. However, it greatly increases the frequency of successful detections.

As in manual staining, the sequence of antibodies can significantly increase or decrease the level of specific signals and non-specific background. This can be explained by the differences in the stability of the epitopes, the conformation of the target proteins, the effect of antigens' proximity and/or other less clear factors related to the execution of experiments with automated machines. Researchers must still determine the optimal sequence of antibodies through trial and error.

The choice of secondary antibodies should also be adjusted to eliminate possible non-specific binding. For example, widely used goat anti-rabbit secondary antibodies would recognize rabbit anti-mouse antibodies and produce non-specific staining in multiplex IF experiments that include primary antibodies produced in goat. Again, this is an important aspect of any multi-antigen staining and often is neglected as the major attention is focused on the primary antibody specificity and clonality.

The staining approach we describe in this article allows researchers to utilize both paraffin sections and cryosections. Our experience suggests that IF staining could be carried with paraffin sections from clinical specimens, even archived. That widens opportunities for assessment of a large number of tissues with well-preserved morphology, including clinically important human tissue specimens. While we presented here IF staining of formalin fixed paraffin-embedded tissues, using the same protocols we have successfully stained frozen tissues, as well as fixed cells.

Another key aspect of our method is that the stainings are performed consecutively. That allows analysis of the results from each staining step to be performed sequentially. The most appropriate marker for the following staining could thus be identified. Importantly, slides can be digitally scanned and the data stored at any step of the sequential staining. As we demonstrate, the presented method of automated IF staining is a powerful and efficient tool that enables reproducible, reliable, high quality staining data to be obtained. Additionally, IF signals can be accurately and reliably quantitated and correlated to antigen expression levels. Such results can bring immeasurable contribution to basic and clinical research. Localization of multiple markers in the same tissue section provides unique insight about spatial, cell – type and even organelle – type specific distribution of molecules of interest. It allows deeper understanding of the tissue during development, in normal adult state, in disease and after treatment.

## Supplementary Material

Supplementary Information Supplementary information

## Figures and Tables

**Table 1:** Antibodies used in staining presented in article

Antibody name	Concentration	Producing company	Catalog number
Primary antibodies			
CD3 (rabbit)	1.2 ug/ml	DAKO	A0452
CD31 (mouse)	5 ug/ml	DAKO	M0823
CD31 (rat)	1 ug/ml	Dia-Nova	DIA-310
CD4 (mouse)	1.2 ug/ml	Abcam	Ab846
CD8 (mouse)	5 ug/ml	DAKO	M7103
c-Myc (rabbit)	2 ug/ml	Epitomics	P01106
E-Cadherin (mouse)	2.5 ug/ml	BD	610181
Ki67 (rabbit)	0.4 ug/ml	Vector	VP-K451
Laminin (rabbit)	2 ug/ml	Sigma	L-9393
Lyve1 (goat)	1 ug/ml	R&D Systems	AF2125
N-Cadherin (mouse)	1 ug/ml	BD Biosciences	610920
PCNA (mouse)	0.25 ug/ml	DAKO	M0879
VASA (rabbit)	2.5 ug/ml	Abcam	ab13840
Vimentin (guinea pig)	0.1 ug/ml	Progen	GP53
Secondary antibodies			
Anti-rabbit (goat)	1:200	Vector	PK4000
Anti-mouse (horse, MOM kit)	1:250	Vector	MKB2225B
Anti-goat (rabbit)	1:200	Vector	PK4004
Anti-rat (rabbit or goat)	1:200	Vector	PK4001/BA9400
Anti-guinea pig (goat)	1:200	Vector	BA7000
Anti-rat-Alexa488 (goat)	1:200	Molecular Probes	A11006
