# Supplementary material for: Machine-based method for multiplex in situ molecular characterization of tissues by immunofluorescence detection
Source: Sci Rep. 2015 Mar 31;5:9534. doi: 10.1038/srep09534 (PMC4821037; doi:10.1038/srep09534)
Supplement: Supplementary Information [file srep09534-s1.pdf]

Supplemental material for the manuscript:

Machine-based method for multiplex *in situ* molecular characterization of tissues by immunofluorescence detection

Dmitry Yarilin, Ke Xu, Mesruh Turkekul, Ning Fan, Yevgeniy Romin, Sho Fijisawa, Afsar Barlas and Katia Manova-Todorova*

Supplemental Table

Successful combinations of antibodies

1st antibody	2nd antibody	3rd antibody	4th antibody
CD31 (mouse)	Vimentin (guinea pig)	E-cadherin (mouse)	PCNA (mouse)
CD31 (rat)	Lyve1 (goat)	E-cadherin (mouse)	Ki67 (rabbit)
c-Myc (rabbit)	CD31 (rat)	pS6R (rabbit)	E-cadherin (mouse)
Meca32 (rat)	Lyve1 (goat)	E-cadherin (mouse)	Ki67 (rabbit)
pMAPK (rabbit)	CD31 (mouse)	Vimentin (guinea pig)	Ki67 (rabbit)

1st antibody	2nd antibody	3rd antibody
CD3 (rabbit)	Olig-2 (rabbit)	ClCaspase3 (rabbit)
CD31 (mouse/rat)	Vimentin (guinea pig)	PCNA (mouse)
CD4 (mouse)	FoxP3 (rat)	CD8 (mouse)
CD4 (mouse)	CD3 (mouse)	CD8 (mouse)
CD4 (mouse)	CD8 (mouse)	FoxP3 (rat)
CD45 (mouse)	AR (rabbit)	Pankeratin (mouse)
CD45 (mouse)	CD3 (rabbit)	B220 (rat)
c-kit (rabbit)	pS6R (rabbit)	PLZF (mouse)
GFP (chicken)	GFAP (rabbit)	Ki67 (rabbit)

GFP (chicken)	Meca32 (rat)	Collagen IV (rabbit)
Meca32 (rat)	Lyve1 (goat)	E-cadherin (mouse)
Meca32 (rat)	Lyve1 (goat)	Ki67 (rabbit)
PCNA (mouse)	pS6R (rabbit)	PLZF (mouse)
PCNA (mouse)	P27 (mouse)	PLZF (mouse)
pHH3 (rabbit)	ClCaspase3 (rabbit)	BrdU (mouse)
PLZF (mouse)	PCNA (mouse)	VASA (rabbit)
pMAPK (rabbit)	CD31 (rat)	Vimentin (guinea pig)
pStat3 (rabbit)	GFP (chicken)	GFAP (rabbit)
pStat3 (rabbit)	GFP (chicken)	Iba1 (rabbit)
SLUG2 (rabbit)	B-catenin (rabbit)	Vimentin (guinea pig)
VASA (rabbit)	Laminin (rabbit)	Ki67 (rabbit)
VASA (rabbit)	Ki67 (rabbit)	P27 (mouse)
ZO1 (mouse)	Vimentin (guinea pig)	Ki67 (rabbit)
ZO1 (mouse)	Vimentin (guinea pig)	AR (rabbit)

1st antibody	2nd antibody
Androgen receptor (rabbit)	Pankeratin (mouse)
Beclin	LC3b (rabbit)
Desmin (mouse)	Tex14 (rabbit)
Cathepsin X (goat)	Iba1 (rabbit)
Cathepsin X (goat)	CD68 (mouse)
Cathepsin X (goat)	Lamp2 (rat)
CD3 (rabbit)	B220 (rat)
CD3 (rabbit)	FoxP3 (rat)

1st antibody	2nd antibody
Androgen receptor (rabbit)	CD45 (mouse)
DAZL (rabbit)	PLZF (mouse)
E-cadherin (mouse)	pStat3 (rabbit)
E-cadherin (mouse)	N-cadherin (mouse)
GFP (chicken)	SMA (mouse)
GFP (chicken)	Meca32 (rat)
GFP (chicken)	KI67 (rabbit)
GFP (chicken)	Iba1 (rabbit)

CD3 (rabbit)	GFAP (rabbit)
CD3 (rabbit)	Neun (mouse)
CD31 (mouse)	pMAPK (rabbit)
CD31 (mouse/rat)	Vimentin (guinea pig)
CD45 (mouse)	CD3 (rabbit)
ClCasp3 (rabbit)	pHH3 (rabbit)
ClCasp3 (rabbit)	pH2AX (mouse)
ClCasp3 (rabbit)	Ki67 (rabbit)
c-Myc (rabbit)	BrdU (mouse)
c-Myc (rabbit)	CD31 (mouse)
DAZL (rabbit)	PCNA (mouse)
B-Tubulin IV (mouse)	Delta-Tubulin (mouse)

Id1 (rabbit)	Olig2 (rabbit)
IF1 (mouse)	OxPhos (mouse)
Ki67 (rabbit)	ClCaspase3 (rabbit)
Ki67 (rabbit)	GFAP (rabbit)
Lamp2 (rat)	LC3b (rabbit)
Lyve1 (goat)	Podoplanin (hamster)
Meca32 (rat)	Lyve1 (goat)
PCNA (mouse)	E-cadherin (mouse)
PCNA (mouse)	Meca32 (rat)
PLZF (mouse)	Vimentin (guinea pig)
VASA (rabbit)	Ki67 (rabbit)
Vimentin (guinea pig)	P27 (mouse)
